# The association between sexual dysfunctions and severity of symptoms in patients with chronic spontaneous urticaria

**DOI:** 10.1186/s13223-018-0244-y

**Published:** 2018-05-16

**Authors:** Agnieszka Skrzypulec-Frankel, Katarzyna Bieniek, Alicja Kasperska-Zając

**Affiliations:** Clinical Department of Internal Diseases, Dermatology and Allergology / European Center for Diagnosis and Treatment of Urticaria (GA2LEN UCARE Network), SMDZ in Zabrze, Medical University of Silesia in Katowice, ul. M. Curie-Skłodowskiej 10, 41-800 Zabrze, Poland

Dear Editor,

We would like to draw attention to the problems of sexual functions and mood disorders in chronic urticarial patients.

Urticaria is a common skin disease. The main symptoms of urticaria are raised above the skin surface red, swollen areas, which are often accompanied by itching. Treatment of chronic urticaria is focused on achieving an adequate level of remission, by addressing the potential causes of the disease. Unfortunately, difficulties in achieving a satisfying treatment results are being seen more and more often. We observe increasing number of patients resistant to the standard treatment regimens. They require extended diagnostics, followed by long-term treatment process, which can by unsuccessful [1]. Skin conditions determine the emotional and psychological aspects of life and have potential to impair human’s functioning in society, including social contacts, work and sexual activity. As an example, chronic pruritus may cause stress, fatigue and problems with sleeping. All of the above have been reported to also have negative effects on quality of life of urticaria patients [2, 3]. The need of taking care of both physical and psychological manifestations of the disease is becoming increasingly visible in modern approach to medicine. To the best of our knowledge, there is only a small number of studies available, in which the sexual functions of chronic urticaria patients were examined [2]. Therefore, the aim of this study was to evaluate the incidence and severity of sexual dysfunctions, mood disorders and their association with severity of skin symptoms in patients with chronic spontaneous urticaria (CSU).

95 patients (49 female and 46 male) of varying CSU activity were examined with weekly Urticaria Activity Score (UAS7) and included into study group [1]. Additionally, both the above group and 92 healthy people from general population that formed control group (45 male and 47 female) were examined with Depression Anxiety Stress Scale (DASS 21), Female Sexual Function Index (FSFI)—female participants and International Index of Erectile Function (IIEF)—male participants [4]. Questions regarding socio-demographic status, disease history and basic anthropometric data were asked in addition to above questionnaires. The main inclusion criteria were age between 18 and 45, clinical diagnosis of CSU (in the study group), informed consent for the participation in the study. The age upper-limit of 45 was established to eliminate potential impact of hormonal changes in perimenopause period on sexual and mood disorders, known from available literature [5]. Exclusion criteria consisted of pregnancy, psychiatric diseases, major diseases that could potentially affect sexual functions, use of mood affecting drugs. Study was approved to be performed by the Bioethical Committee of the Medical University of Silesia. Non-parametric Mann–Whitney U and Spearman tests were used in statistical analysis.

Participants mean age was 35 ± 5.13 in the study group and 33 ± 4.28 in the control group. The majority of study participants were married (70% in study group and 74% in control group). Higher incidence of sexual dysfunctions and mood disorders was found in both female and male CSU patients, compared to general population. The mean result of DASS 21 depression scale was 7.13 ± 4.12 in the study group compared to 4.12 ± 2.65 in the control group and the difference was statistically significant (p < 0.001). Similarly, there was a significant difference between groups in DASS 21 stress questionnaire: 11.24 ± 6.11 in study group, compared to 5.89 ± 3.67 in control group (p < 0.001). No significant difference was found for the anxiety part of DASS 21 questionnaire, with the result of 4.21 ± 2.59 in study group and 3.05 ± 1.96 in control group. Additionally, comparison of sexual functions shown statistically significant higher occurrence of dysfunctions in both male and female questionnaires: FSFI mean results were 27.11 ± 5.1 in study group vs. 30.48 ± 4.25 in control group (p < 0.05) and IIEF 64.28 ± 5.1 vs. 67.63 ± 4.25 accordingly (p < 0.05). Statistically significant differences that contributed to the overall result were found in following questionnaires domains: FSFI—desire, arousal, orgasm and satisfaction; IIEF—erectile function, orgasmic function and sexual desire. Furthermore, statistically significant correlation between the severity of both mood and sexual dysfunctions (DASS 21, FSFI, IIEF) and the severity of urticaria skin symptoms (UAS7) was found (p < 0.05).

To our knowledge, this is the first study approaching sexuality of female and male CSU patients stratified by the disease severity. The potential limitation of this study is that it does not address the sequence of events and does not explain whether CSU impair sexual function and, subsequently, lead to depression and stress or the other way around. A prospective study would be required to answer those questions. Additionally, including partners of CSU patients in further studies could contribute to better understanding of the nature of sexual dysfunctions. Nevertheless, the above results suggest that sexual dysfunctions are more frequent in CSU patients as compared with healthy subjects and are associated with the severity of urticaria symptoms. The main question emerging from this study is how we could better address holistic approach to the treatment of chronic urticaria? Taking into consideration the negative effects of sexual dysfunction on the quality of life [6], we conclude that the need of providing high quality psychological and sexological help to CSU patients seems to be very important.
